# Motivations of nurses who re-entered during the COVID-19 pandemic to either stay or leave nursing practice: A follow-up interview study

**DOI:** 10.1016/j.ijnsa.2026.100707

**Published:** 2026-10-12

**Authors:** Aafke P.M. Arts, Anneke L. Francke, Sofie A. Noorland

**Affiliations:** a The Netherlands Institute for Health Services Research (Nivel), P.O. Box 1568, Utrecht, 3500 BN, the Netherlands; b Nursing Science, program in Clinical Health Sciences, University Medical Centre Utrecht, Utrecht University, Universiteitsweg 98, Utrecht, 3584 CG, the Netherlands; c Faculty of Behavioural and Social Sciences, University of Groningen, Groningen, the Netherlands

**Keywords:** COVID-19, Nursing staff, Pandemics, Personnel turnover, Health workforce, Interviews, Qualitative research

## Abstract

**Background:**

During the COVID-19 pandemic, former nurses were encouraged to return to the nursing profession to ease pressure on the healthcare system. Retaining re-entering nurses in the long term would offer an opportunity to mitigate the persistent nursing shortages.

**Objective:**

To provide insights into the motivations of nurses in the Netherlands who re-entered nursing during the first wave of the COVID-19 pandemic to either remain in or leave nursing again.

**Design:**

A qualitative research design using semi-structured interviews.

**Setting:**

The study was conducted in the Netherlands at various healthcare settings, including hospitals, nursing homes, rehabilitation centres, home care, COVID-19 units, and public health vaccination sites.

**Participants:**

Nurses who had re-entered nursing during the COVID-19 pandemic participated in this follow-up study.

**Methods:**

Thirteen individual semi-structured interviews were conducted in 2025. Verbatim interview transcripts were analysed using reflexive thematic analysis.

**Results:**

The re-entering nurses were categorised into two groups: three 'stayers', who were still working in nursing in 2025, and 10 'leavers', who had left nursing again. For stayers, the following main themes emerged from the analysis: *a meaningful job, professional development opportunities combined with favourable working conditions*, and *support from managers and employers*. For leavers, the main themes identified were: *lack of professional development opportunities, unfavourable working conditions, lack of support from employers and managers, limited professional autonomy and recognition*, and *the intensive trajectory for re-registration for registered nurses.*

**Conclusions:**

Most motivations of re-entering nurses to either remain in or leave nursing reflected broader challenges affecting nurse retention. Addressing these challenges may help to retain re-entering nurses.

**What is already known**

• During the COVID-19 pandemic, inactive nurses were actively encouraged to re-enter nursing to address urgent workforce shortages.
• Re-entering nurses reported specific needs to support their return during the pandemic, including clear role descriptions, personalised training focused on pandemic-related skills, and responsive mentorship structures.
• The return of inactive nurses created opportunities for workforce retention; however, existing literature has primarily focused on re-entry experiences, with a lack of insights into the long-term retention of this group of re-entering nurses.

**What this paper adds**

• The decision to remain in or leave nursing among nurses who re-entered the profession during the COVID-19 pandemic in the Netherlands was primarily motivated by organisational and structural aspects, including professional development opportunities, support from managers and employers, working conditions, professional autonomy, and recognition and re-registration procedures.
• COVID-19 re-entry initiatives may have represented a missed opportunity for long-term nurse retention, as many re-entering nurses received little encouragement or support to remain in the nursing profession from managers and employers.
• Strategies that may improve the long-term retention of re-entering nurses after a pandemic include proactive follow-up of re-entering nurses, greater access to professional development and career opportunities, supportive and autonomous working environments, and tailored re-registration pathways.

## 1 Background

The global nursing workforce is projected to face a shortage of 4.5 million nurses by 2030 (Boniol et al., 2022). The COVID-19 pandemic exacerbated this issue through increased patient volumes, staff infections and nurse attrition, which placed additional pressure on healthcare systems (Fernandez et al., 2020; Nashwan et al., 2021; Turale et al., 2020). In response, strategies were implemented across Western countries to bolster the nursing workforce, including initiatives to reintegrate former nurses (Anesi et al., 2020; Castner et al., 2021; Lücker et al., 2023). For example, in the United States, public figures called on former nurses to return to practice during the pandemic (Andrews, 2020), supported by executive orders that waived licence reactivation requirements to reduce administrative barriers (Alexander, 2021; Andrews, 2020). In Germany, various authorities and organisations similarly appealed to former nurses, often referred to as the “nursing reserve”, to register and assist in managing the crisis (Lücker et al., 2022). In the Netherlands, a comparable approach was adopted through the launch of the national campaign *Extra Hands for Healthcare* in 2020, an initiative jointly established by the government, labour unions, and healthcare organisations (Dekker et al., 2021). In March 2020, the Dutch government also introduced a temporary emergency measure that allowed former nurses whose registration in the Dutch Individual Healthcare Professions Register (BIG register) had expired relatively recently (i.e., on or after 1 January 2016) to return to nursing without immediate re-registration. This was particularly relevant because registration in this register is a legal requirement for nurses to practise as registered healthcare professionals in the Netherlands. The measure remained in place until 1 August 2022, after which nurses were again required to complete the regular re-registration procedure to continue practising as registered nurses (Ministry of Health, Welfare and Sport, 2020, 2025). Although the exact numbers are unknown, the dashboard of the campaign indicates that thousands of former nurses in the Netherlands re-entered nursing during the first year of the pandemic (Dekker et al., 2021).

The motivations of these nurses to return to practice during the COVID-19 pandemic varied. Reasons for re-entering included a sense of duty to contribute to managing the pandemic, a sense of belonging to the nursing profession, a desire to support former colleagues, and the extent to which nurses felt needed in their occupation prior to re-entry (Lücker et al., 2023). How nurses experienced their re-entry was explored by Noorland et al. (2021) in 2020, who interviewed re-entering nurses in the Netherlands about their experiences and needs shortly after their return. Nurses described working during the pandemic as challenging, particularly due to lack of clarity regarding roles and responsibilities. However, positive team dynamics offered a sense of solidarity and support, and conversations with colleagues helped them cope with these challenges (Noorland et al., 2021). Furthermore, Noorland et al. (2021) identified several needs to support nurses in continuing to work in nursing, including clear role descriptions, personalised training focusing on pandemic-related skills, and responsive mentorship. Whether these nurses continued working in nursing post-pandemic or intended to do so was at that time unclear.

To date, limited insight exists into the motivations influencing whether nurses who re-entered practice during the pandemic remained in or left the profession post-pandemic. Nevertheless, some researchers have identified factors that were generally important for nurse retention during the COVID-19 pandemic. For instance, Yamamoto et al. (2024) conducted an interview study among re-entering nurses in Japan, identifying enabling factors, such as support and recognition from colleagues and managers and access to on-the-job refresher training. Motivating factors included pride in contributing to society, opportunities for personal growth, positive family dynamics, and financial stability (Yamamoto et al., 2024). In addition, a report by the International Council of Nurses (Buchan and Catton, 2023) highlighted that protection, opportunities for rest and time off, and support for well-being were key factors in supporting and retaining nurses while working during the pandemic. Beyond the pandemic context, Pressley and Garside (2023) identified several factors associated with nurse retention, including job satisfaction, organisational culture, professional relationships, and individual professional, emotional, and psychological factors, such as stress, resilience, autonomy, career growth, and professional values. Other empirical studies and international reviews have further identified limited social support, limited opportunities for self-development and professional advancement, and high workloads as factors discouraging continued employment in nursing (Ejebu et al., 2024; Hung and Lam, 2020; Lyu et al., 2024; Roth et al., 2022).

However, the applicability of the aforementioned factors to nurses who re-entered specifically during the COVID-19 pandemic remains unclear. These nurses represent a unique group, as they returned to nursing under exceptional circumstances, often after years away from clinical care and while working in highly demanding healthcare environments (Akkuş et al., 2022; Irandoost et al., 2022; Lai et al., 2020)*.* These experiences may have provided unique insights into their motivations to remain in or leave nursing, making this group particularly informative to study. Understanding their experiences can provide valuable insights that extend beyond the pandemic context, as healthcare systems around the world continue to face persistent nursing shortages and may increasingly need to rely on former nurses as a potential workforce in the future (Schlosser et al., 2022). Understanding which factors influence retention among re-entered nurses can inform recruitment, onboarding, support programmes, and retention strategies.

Therefore, in 2025, we re-interviewed nurses who had first been interviewed in 2020 by Noorland et al. (2021). These nurses had returned to nursing during the first wave of the COVID-19 pandemic. This follow-up study aimed to explore their motivations to either remain in or leave nursing after the pandemic.

## 2 Methods

### 2.1 Design

We used a qualitative research design with semi-structured interviews for data collection.

### 2.2 Sample and recruitment

A sample of 13 nurses from the Netherlands was interviewed. They were purposively recruited because they previously participated in the aforementioned interview study by Noorland et al. (2021). The inclusion criteria were that nurses had to have returned to nursing during the first wave of the pandemic, between March and June 2020, after not working as a nurse for at least 1 year (Noorland et al., 2021).

In 2025, 16 of the 20 participants from the previous study had a LinkedIn (a professional networking platform) connection with Noorland, the third author of the current article. These 16 nurses were asked in the first quarter of 2025, through a LinkedIn message from Noorland, whether they were interested in participating in a follow-up interview study. In total, 13 of these 16 nurses were willing to participate. Two nurses declined due to time pressures or personal circumstances, while one did not respond. According to their LinkedIn profiles, these three nurses were no longer working in nursing.

### 2.3 Data collection

Individual semi-structured interviews with the 13 participants were conducted between January and April 2025 by the first and last author. The research team consisted of three female researchers with backgrounds in nursing and health sciences and experience in qualitative health research. The third author had conducted the original interview study in 2020 and was familiar with the participants, whereas the first and second authors had no prior relationship with them.

The follow-up interviews took place approximately 5 years after the original study. During this period, no contact occurred between the third author and the participants other than maintaining a LinkedIn connection, which was used solely to re-establish contact for the present study. No personal or professional contact unrelated to the study had taken place. Although familiarity with participants can influence the interview process, the absence of ongoing contact between the two studies and the involvement of researchers without a prior relationship with participants helped mitigate potential influence. Interviews were conducted through co-interviewing by the first and third authors, allowing for complementary perspectives during data collection. During analysis, interpretations and emerging themes were discussed among all authors to critically reflect on and challenge assumptions based on prior knowledge from the 2020 study.

A semi-structured interview guide was developed collaboratively by the research team (Supplementary Material A). The guide was based on the interview guide used by Noorland et al. (2021) and adapted to the aim and research question of the present follow-up study. It included questions about participants’ professional backgrounds, experiences of re-entering nursing, and motivations for either remaining in or leaving nursing.

Depending on participants’ preferences, interviews were conducted in Dutch by telephone (one interview) or online via Microsoft Teams (12 interviews). All interviews were audio-recorded using Microsoft Teams or an audio recorder. Field notes were made during the interviews to capture notable points and guide further probing of emerging topics. Data collection continued until all participants from the follow-up group had been contacted and, where willing, interviewed. As this study was a follow-up of an existing study, no additional participants were recruited; all participants were drawn from the original study. Interview excerpts presented in this paper were translated from Dutch into English for reporting purposes.

### 2.4 Analysis

All interviews were transcribed verbatim. In addition, the researchers made brief summaries of each transcript to capture the aspects that stood out most in relation to the research question. Data were analysed iteratively, with preliminary analysis of early interviews informing subsequent interviews by identifying topics that required further exploration (DiCicco‐Bloom and Crabtree, 2006).

This process was embedded in the six steps of reflexive thematic analysis (Braun and Clarke, 2006; Byrne, 2022). First, all transcripts and summaries of the interviews were read repeatedly to familiarise the researchers with the data. Second, relevant segments were inductively coded using MAXQDA24 (2024). Third, codes that were related were grouped into draft themes. In the fourth step, these themes were reviewed and refined by re-examining the coded segments and the full interview transcripts. A thematic map was then drafted to visualise the themes and relationships between them. In the fifth step, the final themes were labelled and defined, and the thematic map was further refined. The sixth and final step concerned reporting the results in this paper (Braun and Clarke, 2006; Byrne, 2022).

### 2.5 Trustworthiness of the study

To enhance the trustworthiness of the study, more specifically credibility, transferability, dependability and confirmability, the following strategies were used:

*Credibility* concerns the extent to which the findings accurately represent participants’ experiences (Lincoln and Guba, 1985). We enhanced credibility through researcher triangulation. The first author analysed all 13 transcripts, while five transcripts were independently analysed by at least one of the other two authors. Subsequently, all authors collaboratively reviewed the interim and final analysis, ensuring that the themes accurately reflected the interview data (Nowell et al., 2017).

*Transferability* refers to the extent to which readers can judge whether the findings may apply to other contexts (Lincoln and Guba, 1985). We supported transferability by providing information about the professional backgrounds of the participants and by documenting the sampling criteria and strategy.

*Dependability* concerns whether the research process is logical, traceable, and clearly documented (Lincoln and Guba, 1985). Dependability was strengthened using MAXQDA24, which supported a systematic and traceable coding process by enabling structured organisation of the interview transcripts (Encisoet al., 2024; MAXQDA, 2024). In addition, analytic decisions made throughout the process were documented in an audit trail (Carcary, 2021).

*Confirmability* refers to the extent to which the findings are grounded in the data rather than in researcher bias. We enhanced confirmability by including verbatim quotations from participants in the Results section. Furthermore, the development of several thematic maps helped to visualise the main themes and their interrelationships.

### 2.6 Ethical considerations

Participation in the interviews was voluntary and informed consent was obtained beforehand. The study adhered to the Code of Conduct for Health Research (Commissie Regelgeving Onderzoek, 2022) and the Declaration of Helsinki (World Medical Association, 2024). The study also complied with the European General Data Protection Regulation (Schultz and Hennis-Plasschaert, 2016), and all transcripts were anonymised by removing personal and organisational identifiers. Interview transcripts and audio-recordings were securely stored in a restricted-access folder accessible only to the researchers.

This study did not fall under the scope of the Dutch Medical Research Involving Human Subjects Act, as participants were neither subjected to study procedures nor required to follow behavioural rules instructed by the researchers. Therefore, formal ethical approval was not required (Dutch Medical Research Involving Human Subjects Act; see https://english.ccmo.nl/investigators/guide-to-the-review-procedure/does-my-research-fall-within-the-scope-of-the-wmo; Centrale Commissie Mensgebonden Onderzoek, n.d.).

## 3 Results

### 3.1 Background information

Table 1 presents the characteristics of the 13 participants. The interviews lasted between 36 and 71 min (mean: 52 min). During their re-entry into nursing, participants worked in various, and sometimes multiple, healthcare settings. Table 1 presents the setting in which participants spent the majority of their re-entry period. Several participants also worked in additional settings during the pandemic, although for shorter periods, such as public health vaccination sites. The duration of participants’ absence from nursing prior to re-entry ranged from 1 year to 28 years. Their previous work experience as nurses before re-entry ranged from 1 to 26 years.

**Table 1:** Characteristics.

	N
**Age (*years; n*** **=** **13)**	
30 – 39	1
40 – 49	5
50 – 60	7
**Current job status (*n*** **=** **13)**	
Working in nursing	3
No longer working in nursing	10
**Setting during majority of re-entry (*n*** **=** **13)**	
Hospital	3
Home care	1
Nursing home	3
Newly established COVID-19 unit	6
**Experience in nursing prior to re-entry (*years; n*** **=** **13)**	
>0 ≤ 5	2
>5 ≤ 10	6
>10 ≤ 15	1
>15	4
**Time out of nursing prior to re-entry (*years; n*** **=** **13)**	
≤ 5	2
>5 ≤ 10	5
>10 ≤ 15	1
>15	5

At the time of the interview in 2025, 10 of the 13 participants were no longer working in nursing. Following their re-entry in 2020, these participants had worked in nursing during the COVID-19 pandemic for at least 1.5 months.

### 3.2 Thematic map

The main themes arising from the analysis are displayed in the thematic map in Fig. 1. The thematic map illustrates the motivations underlying nurses’ decisions to remain in or leave nursing after their re-entry. The map distinguishes between participants who remained in nursing after re-entering and were still working in nursing at the time of the study (‘stayers’) and those who left nursing again (‘leavers’). The main motivations for staying or leaving are described in the following sections.

**Fig. 1:**
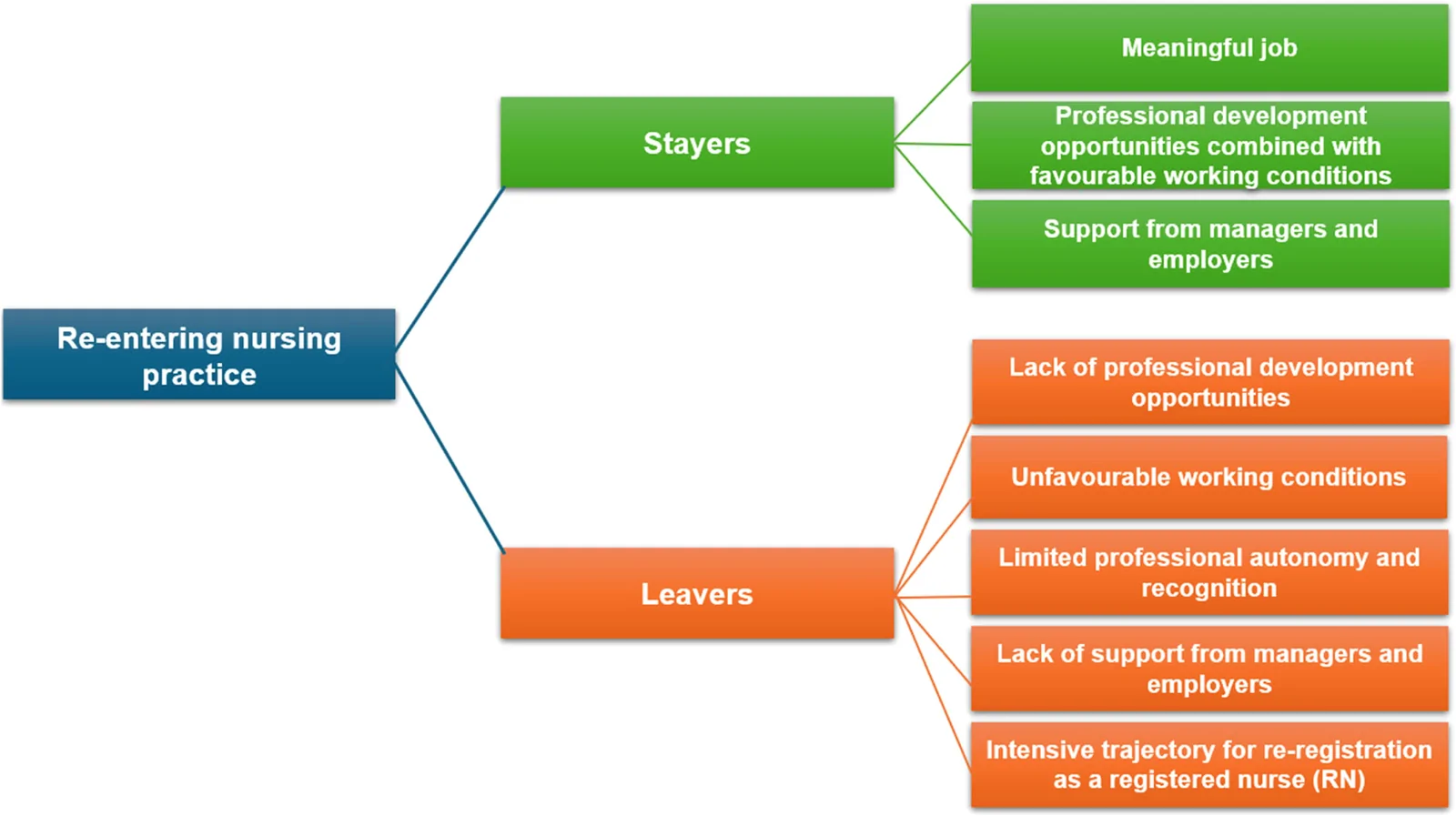
Thematic map.

### 3.3 Motivations of stayers

Three of the 13 participants were classified as ‘stayers’. Like all participants, they had re-entered nursing during the first wave of the pandemic and were still working in nursing at the time of this study. All three nurses initially returned with the intention of supporting the healthcare system during the pandemic but ultimately decided to remain in the profession. For one stayer, the re-entry period also provided an opportunity to rediscover nursing and explore whether the profession had regained its appeal. Although this participant initially returned to her original non-nursing profession after re-entering nursing during the pandemic, she ultimately decided to remain in nursing permanently, concluding that it was the career she preferred.

#### 3.3.1 Meaningful job

All three stayers reported that rediscovering the meaning and value of nursing contributed to their decision to stay. The stayers emphasised the valuable nature of nurse-patient interactions, which was absent in their former non-clinical roles. For instance, through interacting with and helping patients, all three stayers felt they had a meaningful impact, motivating them to continue working after their re-entry. Gratitude expressed by patients and their families for the care provided strongly contributed to this decision. Re-experiencing the meaningfulness of working as a nurse reignited the stayers’ passion for care, which they considered essential for remaining in nursing. Furthermore, one stayer suggested that without a genuine passion for care, nurses would never consider permanently returning to nursing.

*“I found the contact with clients incredibly enjoyable. I realized once again how much I genuinely enjoy working with people. Of course, I already knew that, but I mainly dealt with employees, and that’s a different kind of person after all.”* – ID 4, nurse with 10 years of nursing experience and 16 years of absence from nursing

*“I think it’s different for everyone, but you need to have a heart for healthcare. Otherwise, you won’t do it.”* – ID 8, nurse with 18 years of nursing experience and 3 years of absence from nursing

#### 3.3.2 Professional development opportunities combined with favourable working conditions

Professional development opportunities contributed to stayers’ decisions to remain working in nursing. Around the time of their re-entry, all three stayers described a desire for professional growth and identified opportunities to further develop their careers within nursing. Two of the three stayers transitioned post-pandemic into the roles of advanced practice nurse or general practice nurse. The third stayer pursued a new professional challenge by transitioning to a nursing role in a healthcare setting that was new for her.

*“When I returned to work in the intensive care unit, I realized again why I had left. The irregular hours, but also the desire to do more in policy and management… That’s why I really wanted to become an advanced practice nurse.”* – ID 1, nurse with 9 years of nursing experience and 1 year of absence from nursing

A strong ambition to broaden their scope of practice and participate more actively in policy and decision-making fuelled all these stayers’ motivation to remain working in practice. Opportunities to transition to new roles and sectors within nursing provided possibilities for further professional development and were perceived by stayers as aligning with their professional ambitions. Additionally, the two stayers who transitioned into new nursing roles described that their new roles addressed some of the challenges they had previously encountered in their nursing positions; e.g. irregular shifts that they experienced as physically demanding and difficult to balance with family life. According to these two stayers, their new positions as advanced practice nurse or general practice nurse offered more regular working hours, and greater influence in policymaking. Consequently, professional development opportunities simultaneously supported both career advancement and improved working conditions, thereby contributing to re-entering nurses’ retention.

*“But yes, I really want to continue developing myself. I wanted to pursue a master’s degree. I simply wanted to have a say in policymaking.”* – ID 1, nurse with 9 years of nursing experience and 1 year of absence from nursing

*“So yes, the fact that you can continue doing half of the work as before but now with regular hours was very appealing to me****.***” – ID 8, nurse with 18 years of nursing experience and 3 years of absence from nursing

#### 3.3.3 Support from managers and employers

Employers’ and managers’ support also motivated all stayers to continue working in nursing post-pandemic. Two stayers frequently experienced supportive engagement from employers and managers after their return, encouraging long-term retention. Employers expressed appreciation and their desire to retain the nurses. All stayers were proactively approached, resulting in job offers. When stayers initially declined these offers, employers emphasised the offer would remain open. This prompted them to reconsider long-term plans.

*“Yes, they did ask if staying here to work might be something for me. I replied that the plan was to return to education. But they said, ‘If you change your mind, the door is always open’”.* – ID 8, nurse with 18 years of nursing experience and 3 years of absence from nursing

All stayers felt warmly welcomed after their re-entry into nursing by managers and soon were offered a permanent contract. According to all stayers, employers made visible efforts to facilitate continued employment in nursing and enhance its appeal, which these stayers perceived as motivating.

“*I was immediately offered a permanent contract. They were very pleased with me.”* – ID 8, nurse with 18 years of nursing experience and 3 years of absence from nursing

“*So, I just wrote a letter explaining that I wasn’t yet registered as a nurse, but that I found it very interesting. They were really pleased with my application and were willing to wait.”* – ID 4, nurse with 10 years of nursing experience and 16 years of absence from nursing

### 3.4 Motivations of leavers

Ten of the 13 participants were ‘leavers’ who all had re-entered nursing during the first wave of the pandemic but subsequently left nursing again as the pandemic progressed. These nurses had primarily re-entered nursing to help others, without having a strong intention to work in practice on the longer term.

#### 3.4.1 Lack of professional development opportunities

A lack of development opportunities was a main motive for leavers to quit nursing again. In pursuit of professional growth, they had advanced in other fields outside nursing before re-entering. After their return, leavers again felt that working in nursing in the long term did not align with their professional aspirations.

*“The fact that you’ve already developed further and find the profession less fitting makes you prefer to continue developing in another area where you’re already active.”* – ID 10, nurse with 6 years of nursing experience and 7 years of absence from nursing

Leavers did not pursue professional development within nursing, as they saw no available opportunities. They were never approached by their employers or managers regarding such opportunities, leading them to assume none existed. Development roles’ lack of visibility, coined by leavers as a “branding issue”, was a persistent barrier to nurse retention.

“*That you can achieve all of that as a nurse… You can truly become anything you want within this field. (…) I believe that is something truly unique to this sector. Yes, in my opinion, that’s exactly where the branding really falls short. (…) The opportunities within healthcare are incredibly diverse, but I think they are far too rarely made visible.” –* ID 11, nurse with 18 years of nursing experience and 12 years of absence from nursing

#### 3.4.2 Unfavourable working conditions

Additionally, unfavourable working conditions emerged as a reason for not remaining in nursing after re-entering. Compared to their current jobs at the time of the interview in 2025, leavers considered that the benefits of working in nursing did not outweigh the drawbacks. The leavers also described the irregular shifts as physically demanding and difficult to combine with family life. This was particularly evident when compared to their current non-nursing jobs, where they can plan their working days more independently, avoid strict holiday policies (requiring staff to coordinate their leave to ensure minimum staffing levels are maintained), and take time off more freely. Lastly, although salary differences were not the primary reason for leaving nursing, several participants nevertheless mentioned them as a secondary but meaningful barrier. Given their current salaries, continuing to work in nursing would have resulted in a financial setback, requiring lifestyle adjustments that leavers did not consider worthwhile.

*“Well, it’s definitely a combination of irregular shifts and having young children at home. That’s tough. The job itself is, of course, demanding, but it’s really the combination with your personal life that makes it hard.” –* ID 11, nurse with 18 years of nursing experience and 12 years of absence from nursing

*“But there are even bigger obstacles, you know, like requesting summer vacation in December. Now I have a lot of flexibility. I can organise my entire days and weeks myself.”* – ID 7, nurse with 26 years of nursing experience and 9 years of absence from nursing

*“Yes, I am obviously used to a certain lifestyle and level of income now. So, working as a nurse in direct patient care would create too large a financial gap.”* – ID 3, nurse with 4 years of nursing experience and 20 years of absence from nursing

#### 3.4.3 Limited professional autonomy and recognition

Leavers experienced limited professional autonomy upon re-entering nursing. They saw the healthcare system as a rigid structure where they did not belong in the longer term. This lack of professional autonomy also related to having little influence over work schedules (see ‘Unfavourable working conditions’). In addition, the hierarchical nature of healthcare was a key concern. According to leavers, nurses occupy a low hierarchical position, associated with a lack of recognition and respect. They mentioned that physicians and managers did not always consider nurses as equals in dialogue, which restricted nurses’ professional autonomy, as well as hampered influence in policymaking and implementing changes in practice. This sense of limited autonomy discouraged leavers from remaining in nursing, and they did not see opportunities to contribute meaningfully to improvements in nursing care had they remained in practice.

*“I would like healthcare to be organised differently; otherwise, you wouldn’t do the kind of work I do now. And I often felt that, as a nurse, there was little you could do about it.” –* ID 9, nurse with 15 years of nursing experience and 8 years of absence from nursing

Additionally, leavers associated their limited autonomy with strict daily routines and the protocol-driven nature of nursing care. Leavers perceived the healthcare sector as overly reliant on protocols, which restricted individual initiatives and choices. They missed unpredictability and dynamism, aspects they believed nursing care failed to provide sufficiently.

“*Yes, and that’s why I thought, hey, this is more illness-centred care, it’s very protocol-driven. I’m looking for more autonomy*.” – ID 3, nurse with 4 years of nursing experience and 20 years of absence from nursing

“*The work of a nurse is tied to certain routines. In the morning, you start with washing, then making the beds, and then doing the rounds. So… to be honest, it’s all the same routine every day. Now, I have a lot of freedom in my work, and I really enjoy that.” –* ID 11, nurse with 18 years of nursing experience and 12 years of absence from nursing

#### 3.4.4 Lack of support from managers and employers

After re-entering, employers and managers rarely approached leavers with offers to stay, culminating in a perceived lack of support from managers and employers. Leavers assumed that continued employment was neither expected nor desired. Those who initially remained open to continue working in nursing faced structural barriers, such as having already reached the maximum of three temporary contracts, after which employers are legally obliged under current national legislation to offer a permanent contract. In these cases, employers made no effort to explore alternatives or create opportunities for continued employment, resulting in these leavers leaving nursing. Participants stated that this lack of proactive engagement by employers represented a missed opportunity for organisations to retain re-entering nurses.

*“They didn’t even have a conversation with me. Like, would you want to stay longer? Would you be able to? Or if you stayed longer, what would you need? Would you consider renewing your nursing registration? That conversation just never happened.”* – ID 6, nurse with 10 years of nursing experience and 25 years of absence from nursing

*“Well, it was very simple. We had to leave after three contracts because we had temporary contracts, even though the work was far from finished at the time. So, they just followed the rules.” –* ID 2, nurse with 1 year of nursing experience and 25 years of absence from nursing

#### 3.4.5 Intensive trajectory for re-registration as registered nurse

As described in the introduction section, during the pandemic nurses whose legal registration as a registered nurse (RN) had expired after 1 January 2016 were temporarily allowed to work as a nurse and to return to nursing, without immediate re-registration. This was relevant because registration in the Dutch Individual Healthcare Professions Register (BIG register) is a legal requirement in the Netherlands to work as a practising registered nurse. The temporary measure remained in place until 1 August 2022, after which nurses whose registration had expired were once again required to complete the re-registration procedure in order to practice as registered nurses. Leavers perceived the re-registration process, comprising three exams on knowledge, technical skills, and clinical reasoning, as too burdensome to complete. This perception was associated with high financial costs and time investments to complete the exams. Some of the leavers partly attributed the perceived high time investment to the broad scope of the exams, encompassing nursing in all healthcare sectors rather than the specific sector they worked in. In addition, some participants indicated that they lacked adequate support and guidance to initiate the re-registration process, leaving them uncertain about how to start and what the re-registration trajectory would entail.

*“And another factor was (…) that obtaining this nursing registration required a significant amount of money, which you had to pay for yourself.” –* ID 2, nurse with 1 year of nursing experience and 25 years of absence from nursing

*“Much more tailored, yes. I, I went to university, and then you’re required to take an MBO level 3* [Dutch secondary vocational education; European Qualifications Framework level 3] *exam. I know it might sound like I feel too good for it, but it just wasn’t a good fit.”* – ID 3, nurse with 4 years of nursing experience and 20 years of absence from nursing

## 4 Discussion

In this qualitative study we have described the motivations of nurses who re-entered nursing during the COVID-19 pandemic to either remain in or leave nursing again. Understanding these motivations provides valuable insights into how re-entering nurses can be better supported and retained in the nursing workforce.

We have shown that the motivations of the three nurses who remained in nursing were largely the mirror image of those reported by participating nurses who decided to leave again. Although only three stayers were interviewed, the main motivations identified among this subgroup were consistent and appeared to concern the meaningfulness of their work, opportunities for professional development combined with favourable working conditions, and support from employers and managers to continue working in nursing. In contrast, *leavers* experienced limited opportunities for professional development, perceived working conditions as unfavourable, and reported a lack of support from employers and managers. In addition to this, leavers experienced insufficient professional autonomy and recognition, and considered the re-registration process for registered nurses too burdensome. Hence, professional development opportunities likely played a role in both stayers’ and leavers’ decisions to remain in or leave nursing. This finding aligns with studies conducted in Western countries, where researchers found that limited opportunities for self-development and professional advancement were associated with higher nurse turnover (de Vries et al., 2023; Seller-Boersma et al., 2023).

In our study, stayers also linked professional development opportunities to more favourable working conditions. For example, two of the three stayers described how professional development opportunities provided access to positions with more predictable working hours, which they considered an important advantage. Likewise, leavers identified unfavourable working conditions as an important reason for leaving nursing, a finding that is consistent with Yamamoto et al. (2024). In their qualitative interview study among Japanese re-entering nurses, unfavourable working conditions, including irregular shifts that were difficult to reconcile with family responsibilities and low salaries, were linked to decisions to leave nursing. Although salary was not the sole reason for leaving, leavers in our study perceived that their remuneration did not reflect their experience and qualifications, a finding also supported by other researchers conducting studies of nurses in Western countries (Bäckström et al., 2024; Pyhäjärvi and Söderberg, 2024).

As described, also employer and manager support or lack thereof appears to be a motivation for re-entering nurses to either remain in or to leave nursing again. This finding aligns with previous research conducted before and after the COVID-19 pandemic among nurses in Sweden and Canada. Across these studies, nurse retention was consistently linked to the level of support provided by employers and managers (Bäckström et al., 2024; Chachula et al., 2015; Lögde et al., 2018). Similarly, De Vries et al. (2024) found in a meta-analysis that higher perceived organisational support was associated with lower intention to leave, while supervisor support was identified as a protective factor against nurses’ turnover intention.

Moreover, leavers in our study cited limited professional autonomy as contributing to their departure. They perceived nursing work as overly protocol-driven and felt they barely had decision-making power. Participants felt their limited professional autonomy stemmed from their low hierarchical position and a lack of recognition. In contrast, in the previous interview study by Noorland et al. (2021) during the COVID-19 pandemic, nurses viewed limited professional autonomy associated with a more supportive role positively, as they did not feel comfortable taking on greater responsibility. This difference may reflect the exceptional circumstances of the pandemic. During the crisis phase, reduced responsibility may have been experienced as protective, as re-entering nurses mainly fulfilled supportive roles. In the post-pandemic phase, however, limited autonomy may have become problematic when nurses consider re-establishing themselves as fully recognised professionals. This was in line with findings from a systematic review by de Vries et al. (2023), which found that low decision authority and organisational cynicism negatively affected nurses’ intention to remain in the profession in Western countries.

Stayers reported experiencing their work as meaningful, which motivated them to remain in nursing. Participants described rediscovering the meaning and value of nursing, experiencing meaningful patient interactions, and regaining their passion for care. This is supported by Im and Koh (2023), who found that higher job satisfaction was associated with lower turnover intention among nurses. Similarly, a systematic review by Pressley and Garside (2023) found that job satisfaction, together with feelings of fulfilment and enjoyment, could help nurses remain motivated and better cope with the challenges of nursing work. Moreover, the accounts of the stayers in this study may be interpreted through the lens of Self-Determination Theory, suggesting the presence of autonomous motivation. According to Self-Determination Theory, autonomous motivation is defined as engaging in work because it is personally valued and satisfying, rather than driven by internal or external pressure (Ryan and Deci, 2020; Fernet et al., 2017). Consistent with this perspective, Fernet et al. (2017) found that newly registered Canadian nurses with higher levels of autonomous motivation were less likely to intend to leave either the profession or their organisation. According to Self-Determination Theory, autonomous motivation is fostered through the satisfaction of three basic psychological needs: autonomy (having a sense of choice and control over one’s work), competence (feeling capable and effective), and relatedness (feeling connected to and supported by others) (Ryan and Deci, 2020). Taken together, the accounts of nurses who remained in nursing suggest that they may have experienced greater fulfilment of these basic psychological needs. Specifically, the reported professional autonomy may reflect satisfaction of the need for autonomy. Within the theme “Professional development opportunities combined with favourable working conditions”, opportunities to pursue further education and take on new professional roles may support satisfaction of the need for competence. The theme “Support from managers and employers” may reflect satisfaction of the need for relatedness through feeling connected to and supported by others at work. In contrast, the accounts of nurses who left the profession suggest experiences consistent with frustration of these needs.

Interestingly, we initially expected experiences directly related to working during the COVID-19 pandemic to strongly influence re-entering nurses’ motivations to remain in or leave the profession. However, pandemic-specific experiences did not emerge as a dominant factor. Instead, participants’ motivations largely reflected broader structural challenges affecting nurse retention. One possible explanation is that the pandemic may have amplified existing weaknesses in the nursing work environment. Rather than creating entirely new reasons for leaving, it may have exposed or intensified long-standing challenges affecting retention. Consequently, once the immediate crisis subsided, nurses may have evaluated whether to remain in nursing based on factors that were already relevant before the pandemic.

A recent scoping review and a systematic review on nurse retention before and after the COVID-19 pandemic similarly concluded that many retention challenges observed during the pandemic were not novel but rather reflected pre-existing structural issues (Buckley et al., 2025; De Vries et al., 2024). The reviews, however, also identified several COVID-19-specific factors influencing nurses’ intentions to leave, including fear of infection, concern about transmitting the virus to family members, shortages of and limited confidence in personal protective equipment, and feeling unprepared to care for COVID-19 patients (Buckley et al., 2025; De Vries et al., 2024). Although COVID-19–related motivations were not directly reported in our sample, the relatively low number of stayers may still reflect the broader trend of increased nurse attrition after the pandemic. Enea et al. (2024) found in an international study that higher intention to leave nursing was associated with serving on the frontline during COVID-19, living in the Netherlands, and experiencing limited opportunities for professional development.

The low retention of re-entering nurses in our study is also consistent with evaluations of the Dutch national initiative *Extra Hands for Healthcare*, which aimed to mobilise additional healthcare staff during the pandemic (Dekker et al., 2021). These evaluations indicate that the primary value of the initiative lay in providing temporary “extra hands” during a crisis rather than creating sustainable pathways for long-term re-entry into the profession. Taken together, we suggest that crisis-driven mobilisation alone is insufficient to ensure the long-term retention of re-entering nurses.

Nevertheless, from our results, we indicated that the temporary mobilisation of re-entering nurses during the pandemic in the Netherlands may have represented a missed opportunity for longer-term workforce retention. Several participants reported receiving little guidance regarding the possibility of returning to nursing on a more permanent basis, and continuing in the profession was not consistently encouraged. Moreover, the observation that nurses who remained in nursing reported access to the very conditions that leavers perceived as lacking indicates that retention of re-entering nurses may be achievable if these structural challenges are addressed.

The high number of nurses leaving the profession after the COVID-19 pandemic, combined with the difficulty in retaining re-entering nurses who contributed during the crisis, threatens to undermine efforts to rebuild healthcare systems and may have important implications for preparedness for future pandemics. Apart from increasing pressure on an already stretched workforce, valuable crisis experience and expertise risk being lost, resources essential for responding to future health emergencies. This underscores the urgent need to address structural factors affecting nurse retention, a concern also highlighted by the International Council of Nurses which warns that failure to do so may compromise both health system recovery and preparedness for future health crises (Buchan and Catton, 2023).

## 5 Limitations and strengths

A limitation of this study is the small number of stayers (*n* *=* *3*), and, therefore, no firm conclusions about motivations for remaining in nursing can be drawn. The limited number of stayers also prevented us from establishing thematic saturation within this subgroup (Guest et al., 2020). Nevertheless, the narratives of the three stayers showed consistency and revealed a pattern that mirrored the accounts of leavers. The motivations of stayers to remain in nursing corresponded to the absence of or dissatisfaction with those same aspects among leavers. This convergence across perspectives supports the relevance of the identified factors for understanding nurse retention, although further research is needed to confirm their role in supporting continued employment.

Another limitation concerns participant recruitment. This study was a follow-up of a previous study. LinkedIn was used solely to re-establish contact with participants from the original 2020 study, as no alternative contact details were available. However, four participants could not be contacted. In addition, three other participants (who were all ‘leavers’) did not take up our invitation to be interviewed. Their experiences remain unknown.

A further limitation of this study is that we did not validate the identified themes through member checking among participants. The main reason for this was that (busy) professionals often do not commit to additional rounds of feedback. However, we do not expect that this has led to any major distortion of the results because the main themes can be traced across all interviews. Moreover, various other measures were used to enhance the quality and trustworthiness of the research, including researcher triangulation, peer debriefing, the use of software to support the analysis, and maintaining an audit trail. Furthermore, within the leaver subgroup, thematic saturation was achieved, with no substantially new themes emerging after seven interviews. Together, these measures strengthened the trustworthiness of the findings.

## 6 Implications

Based on this study, several opportunities could be considered to support the sustained employment of nurses after re-entry. Healthcare organisations and managers should recognise re-entry as a potential pathway to long-term retention and proactively discuss with re-entering nurses whether they intend to remain in nursing and what support they need to do so. Such conversations can help identify individual barriers and inform tailored support to facilitate the transition from temporary re-entry to continued employment. However, because this study examined only the perspectives of re-entering nurses, it may have overlooked legitimate reasons employers had for not offering continued employment. Future researchers should examine the experiences and perspectives of employers and managers to provide a more balanced understanding of these employment decisions.

At the organisational and professional levels, attention may also be needed to the structural factors associated with retention identified by nurses who left again. Healthcare organisations, educators, and policymakers could improve access to and visibility of professional development opportunities and career pathways for re-entering nurses. These opportunities may support professional growth while enabling them to move towards working conditions that better fit their circumstances. Supportive team environments are equally important, including recognition and opportunities to exercise professional autonomy. Managers could maintain regular contact with nurses and actively monitor their experiences in everyday practice and identify barriers that may otherwise remain overlooked.

At the level of the profession, opportunities to reduce barriers to re-registration through policy should be explored. We suggest that the current process may discourage re-entry, as former nurses described it as burdensome and expressed a need for a more tailored approach. Re-registration requirements could potentially account for nurses’ previous knowledge and professional experience, as well as the care sector in which they intend to work. Researchers should examine how re-registration requirements can be tailored to reduce unnecessary barriers to re-entry while maintaining standards for safe and competent practice. Given the limited evidence on re-registration as a potential barrier to sustainable re-entry, further research is needed to better understand how the re-registration process influences nurses’ decisions to return to and remain in nursing.

## 7 Conclusions

We identified motivations of nurses who re-entered during the COVID-19 pandemic to either remain in or leave nursing. Motivations to stay included professional development opportunities combined with favourable working conditions, meaningful work, and support and encouragement from employers and managers. In contrast, motivations to leave included limited professional development opportunities, unfavourable working conditions, limited professional autonomy and recognition, a lack of support from employers and managers, and the burdensome re-registration process as an RN. Hence, most motivations to either remain in or leave nursing reflected broader organisational and structural challenges affecting nurse retention. Addressing these issues may help to retain re-entering nurses and contribute to workforce stability and healthcare system resilience.

## Declaration of generative AI and AI-assisted technologies in the manuscript preparation process

During the preparation of this work the author(s) used ChatGPT in order to optimise the language. After using this tool, the authors reviewed and edited the content as needed and take full responsibility for the content of the published article.

## Funding

This research did not receive any specific grant from funding agencies in the public, commercial, or not-for-profit sectors.

## CRediT authorship contribution statement

**Aafke P.M. Arts:** Writing – review & editing, Writing – original draft, Visualization, Validation, Resources, Methodology, Investigation, Formal analysis, Data curation, Conceptualization. **Anneke L. Francke:** Writing – review & editing, Validation, Supervision, Resources, Methodology, Formal analysis, Conceptualization. **Sofie A. Noorland:** Writing – review & editing, Validation, Supervision, Project administration, Methodology, Investigation, Formal analysis, Conceptualization.

## Declaration of competing interest

The authors declare that they have no known competing financial interests or personal relationships that could have appeared to influence the work reported in this paper.
