# Supplementary material for: Motivations of nurses who re-entered during the COVID-19 pandemic to either stay or leave nursing practice: A follow-up interview study
Source: Int J Nurs Stud Adv. 2026 Oct 12;11:100707. doi: 10.1016/j.ijnsa.2026.100707 (PMC13717889; doi:10.1016/j.ijnsa.2026.100707)
Supplement: Supplementary file 1 [file mmc1.docx]

# Supplementary Material A: Interview Guide

| **Introduction** | |
| --- | --- |
| **Introduction** | - Introduce yourself and your role as the researcher. - Thank the participant for taking part in this study. - Answer any questions the participant may have regarding the participant information sheet and informed consent form that were provided prior to the interview. |
| **Purpose of the interview** | - • Brief explanation of the purpose of the interview:  1. The purpose of this interview is to understand your motivations for either continuing or not continuing to work as a nurse after the pandemic. 2. Hereby, we hope to gain a better understanding of the motivations that may contribute to retaining re-entering nurses in the nursing workforce following a pandemic. |
| **Privacy** | - The information collected during this interview will be processed anonymously and used in a scientific article. This means that no personal data or information that could identify you will be included in the article. - You may stop the interview at any time if you no longer wish to participate. |
| **Interview structure** | - I would like to record this interview using Microsoft Teams. Before we begin, I would like to ask for your permission once again to record this interview. **(Start recording)** |
| **Themes** | |
| **Brief reflection on returning to nursing practice during the pandemic** | - We will first briefly discuss the period in which you returned to nursing practice. - What motivated you to return to work as a nurse during the pandemic? How were you approached? - Could you briefly describe how your return to nursing practice took place?   *Probing questions:*   - *Where did your return to nursing practice take place?* - *Could you briefly explain your position and the responsibilities associated with your role?* - How do you reflect on your return to nursing practice now? - Did you experience the different waves of the pandemic differently?   Probing topics and questions:  *Role responsibilities and task description:*   - *Looking back now, how do you reflect on your role and how it was fulfilled as a nurse returning to practice?* - *Did the responsibilities and role description change over time?* - *Looking back on this period, what support do you think - based on your experience - is important regarding task allocation and defining the role of nurses returning to practice during a pandemic?*   *Supervision and support:*   - *Looking back now, how do you reflect on the supervision and support you received at the time as a nurse returning to practice?* - *Looking back on this period, what do you think - based on your experience - is important regarding supervision and support for nurses returning to practice during a pandemic?*   *Training:*   - *If you received training: Looking back now, how do you reflect on the training you received at the time as a returning nurse? What type of training did you receive?* - *If you did not receive training: Looking back now, how do you reflect on this?* - *Looking back on this period, what do you think – based on your experience – is important regarding training and continuing education for nurses returning to practice during a pandemic? What format and topics would be most valuable?*   *Team dynamics:*   - *Looking back now, how do you reflect on the collaboration and team dynamics within the team you worked in during the pandemic?*   *Mental health:*   - *In your experience, did your return to nursing practice during the pandemic affect your mental health during the pandemic? Could you elaborate on this?* - *In your experience, did your return to nursing practice during the pandemic affect your mental health after the pandemic? Could you elaborate on this?*   *Mental health support:*   - *Looking back now, how do you reflect on the availability of mental health support during your return to nursing practice?* - *When looking back on this period, what do you think – based on your experience – is important for supporting the mental health of nurses returning to practice during a pandemic?* - *Did you return to nursing practice during all waves of the pandemic?* - Did you experience the different waves differently? - *Did you return to nursing practice during every wave, or did you stop earlier? What was the reason for this?* - *Were you approached by your employer again during each wave to return to work, or did this happen in another way?* - Did you continue working as a nurse after your return to nursing practice?   *Probing questions:*   - *If not: Where did you start working after your return to nursing practice? What position did you take?* - *If yes: Did you continue working at the organisation where you returned to nursing practice? What is your current position?* |
| **After re-entry** | - We will now discuss the period after your return to nursing practice. - After your return to nursing practice, were you actively approached to continue working at the organisation where you returned to practice? - What were your reasons for either continuing or discontinuing your work as a nurse after your return to nursing practice? Could you elaborate on this? - What were the reasons that led you to leave the healthcare profession before the pandemic?   *Probing questions:*   - *If you stopped working as a nurse: Were these the same reasons that led you to stop working as a nurse in the first place?* - *If you continued working as a nurse: What has changed for you compared to before the pandemic that makes you want to continue working as a nurse now?* - Do you still stand by your decision to either continue or discontinue working as a nurse? Why or why not?   *Probing questions:*   - *If you stopped working as a nurse: Why did you choose your current job instead of continuing in the nursing profession?* - *If you stopped working as a nurse: What would you have needed in order to potentially continue working after your return to nursing practice? Do you think this also applies to other nurses returning to practice?* - What do you think is needed to retain nurses who have returned to practice after a pandemic? - If a similar crisis situation, such as a pandemic, were to occur again, would you consider returning to nursing practice (temporarily) again? Why or why not? |
| **Closing** | |
| **Closing** | - Do you have any additional comments or anything else you would like to add? - If I have any further questions, may I contact you again? - Would you like to receive the publication of the future article? - Thank you for your time and participation. |
| **References** | |
| 1. Noorland SA, Hoekstra T, Kok MO. The experiences and needs of re-entering nurses during the COVID-19 pandemic: A qualitative study. International Journal of Nursing Studies Advances. 2021 Nov; 3: 100043. doi: 10.1016/j.ijnsa.2021.100043. 2. Yamamoto K, Nasu K, Nakayoshi Y, Takase M. Sustaining the nursing workforce - exploring enabling and motivating factors for the retention of returning nurses: a qualitative descriptive design. BMC Nurs. 2024 Apr 17; 23(1): 248. doi: 10.1186/s12912-024-01900-5. 3. Bahlman-van Ooijen W, Malfait S, Huisman-de Waal G, Hafsteinsdóttir TB. Nurses' motivations to leave the nursing profession: A qualitative meta-aggregation. Journal of Advanced Nursing. 2023 Dec;79(12):4455-4471. doi: 10.1111/jan.15696. | |
